# Enhanced identification of membrane transport proteins: a hybrid approach combining ProtBERT-BFD and convolutional neural networks

**DOI:** 10.1515/jib-2022-0055

**Published:** 2023-07-28

**Authors:** Hamed Ghazikhani, Gregory Butler

**Affiliations:** Department of Computer Science and Software Engineering, Concordia University, Montreal, Canada

**Keywords:** neural network, ProtBERT-BFD, protein language model, transformers, transmembrane transport proteins

## Abstract

Transmembrane transport proteins (transporters) play a crucial role in the fundamental cellular processes of all organisms by facilitating the transport of hydrophilic substrates across hydrophobic membranes. Despite the availability of numerous membrane protein sequences, their structures and functions remain largely elusive. Recently, natural language processing (NLP) techniques have shown promise in the analysis of protein sequences. Bidirectional Encoder Representations from Transformers (BERT) is an NLP technique adapted for proteins to learn contextual embeddings of individual amino acids within a protein sequence. Our previous strategy, TooT-BERT-T, differentiated transporters from non-transporters by employing a logistic regression classifier with fine-tuned representations from ProtBERT-BFD. In this study, we expand upon this approach by utilizing representations from ProtBERT, ProtBERT-BFD, and MembraneBERT in combination with classical classifiers. Additionally, we introduce TooT-BERT-CNN-T, a novel method that fine-tunes ProtBERT-BFD and discriminates transporters using a Convolutional Neural Network (CNN). Our experimental results reveal that CNN surpasses traditional classifiers in discriminating transporters from non-transporters, achieving an MCC of 0.89 and an accuracy of 95.1 % on the independent test set. This represents an improvement of 0.03 and 1.11 percentage points compared to TooT-BERT-T, respectively.

## 1 Introduction

**Figure 1:**
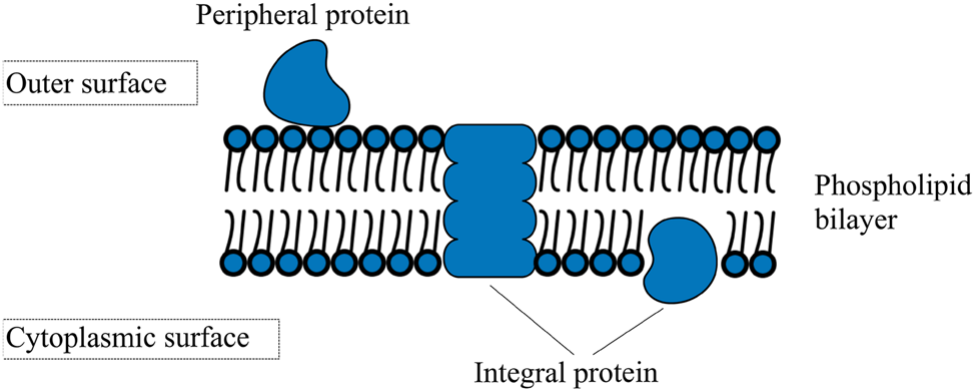
The cell membrane’s structure. This diagram illustrates the structure of the cell membrane, which is composed of two primary components: the lipid bilayer and membrane proteins. Membrane proteins can be surface-bound (also known as peripheral) or integral.

Membrane proteins (Figure 1) play a vital role in various cellular processes and constitute a significant portion of all proteins identified in a cell, accounting for approximately 30 % of the total protein content [1, 2]. These proteins are essential for maintaining the integrity of lipid bilayer membranes, which protect the cell’s interior and are critical for the survival of cellular organisms [3]. Due to the highly restricted diffusion of polar substrates across lipid bilayers, cells have evolved transporter proteins to facilitate the utilization of essential substances from the environment [3]. These substances include anions, cations, vitamins, sugars, nucleosides, amino acids, peptides, bile acids, and porphyrins [3]. Approximately one-third of a cell’s proteins are embedded within biological membranes, and about one-third of these membrane proteins contribute to the transport of molecules across the membrane, highlighting their crucial function in cellular processes [4].

Defective or improperly regulated membrane proteins can disrupt an organism’s cellular functions, leading to diseases [5]. Consequently, examining cell membranes is essential for understanding certain diseases and identifying potential treatments [6]. Membrane proteins have become highly attractive targets in the pharmaceutical industry, with over fifty percent of FDA-approved drugs currently targeting them [6]. This strong connection between membrane proteins and disease treatment further underscores the importance of studying these proteins and their roles in cellular processes.

Despite the availability of numerous membrane protein sequences, largely due to recent genome projects, their structures and functions remain poorly characterized [2]. This is partly due to significant challenges in wet lab characterization, such as crystallization, expression, and structure determination. The gap between the number of available sequences and those with experimentally determined properties hinders progress in biology and drug discovery. Consequently, there is a demand for advanced computational tools capable of distinguishing membrane transport proteins based solely on sequence information. These tools can guide future research and provide insights into protein function, ultimately contributing to our understanding of cellular processes and potential therapeutic targets [7].

**Figure 2:**
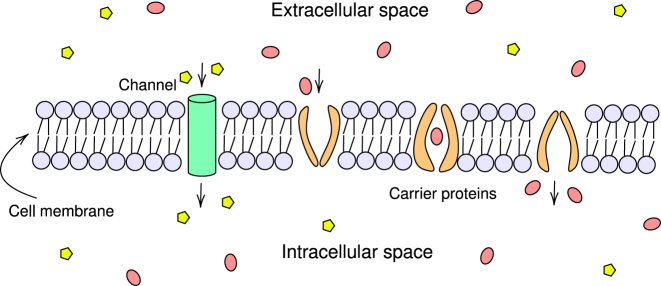
Transport proteins. This diagram illustrates two types of transport proteins, channel and carrier proteins.

*Transmembrane transport proteins* (transporters) (Figure 2) are crucial to the fundamental cellular processes of all organisms [3, 8]. The widespread evolutionary distribution of transporters among prokaryotes and eukaryotes underscores their biological importance [3]. The repertoire of transporters in a specific organism offers valuable insights into its lifestyle and physiology [8]. Until recently, the investigation of membrane transporters primarily focused on examining transporter genes within individual species [8]. However, advancements in genome sequencing now enable researchers to compare transport and other essential cellular functions across a diverse range of organisms spanning all three domains of life [8].

Artificial intelligence models for natural language processing are increasingly adept at understanding and processing language, finding applications in automated speech recognition, translation, intelligent assistants, and text generation [9]. Autoencoders, such as BERT [10], are stacked models trained by corrupting input tokens and attempting to reconstruct the original sentence. While capable of generating text, they are primarily employed to create vector representations for subsequent classification tasks [2].

Computational biologists recognize the potential of these models for simulating biological phenomena. Recent applications of language models include protein function prediction, protein evolution analysis, and protein design [11]. Unsal et al.’s study [12] offers a comprehensive review of natural language models used in protein representation from 2015 to present.

Elnaggar et al. [13] conducted the ProtTrans project, which involved six Transformer-based protein language models (PLMs) trained and compared on secondary structure prediction, subcellular localization, and water solubility tasks. Among these models, ProtBERT and ProtBERT-BFD are two BERT models pre-trained on the UniRef100 database [14] with 216 million protein sequences and the BFD database [15] with 2.1 billion protein sequences, respectively. These models comprise 30 layers of 16 attention head transformer encoders, totaling 420 million parameters. These language models generate a 1024-dimensional vector for each amino acid. MembraneBERT [2] is a fine-tuned ProtBERT-BFD on membrane proteins from the TooT-M dataset [16].

In our previous work, TooT-BERT-T [7], we provided a comprehensive overview of related works on classifying transporters from non-transporters. In TooT-BERT-T, we used a Logistic Regression (LR) classifier in conjunction with ProtBERT-BFD fine-tuned on the transporter dataset of TooT-T [6]. The results demonstrated that the fine-tuned ProtBERT-BFD representation outperforms both MembraneBERT representation and TooT-T on the independent test set, achieving an accuracy of 93.89 % and an MCC of 0.86.

In this study, we employ ProtBERT, ProtBERT-BFD, and MembraneBERT with traditional classifiers, including Support Vector Machine (SVM), Random Forest (RF), k-Nearest Neighbors (kNN), and Feed-Forward Neural Network (FFNN), as well as a deep learning classifier, a Convolutional Neural Network (CNN). TooT-BERT-CNN-T is the fine-tuned representation from ProtBERT-BFD combined with a CNN classifier to distinguish transporters from non-transporters, outperforming all conventional classifiers.

Our contributions are as follows: (1) Analysis of fine-tuned representations from ProtBERT, ProtBERT-BFD, and MembraneBERT. (2) Evaluation of various traditional classifiers and a deep learning classifier. (3) Development of a novel CNN architecture for this task. (4) Proposal of TooT-BERT-CNN-T as a method to discriminate transporters from non-transporters, surpassing all previous approaches.

This paper is organized as follows: The dataset and experimental setup for this study are described in Section 2. The results are compared, analyzed, and discussed in Section 4. Finally, the paper is concluded in Section 5.

## 2 Methods

### 2.1 Dataset

The dataset [17] utilized in this study is widely used by most transporter predictors, including TrSSP [17], SCMMTP [18], Li et al. [19], Ou et al. [20], TooT-T [6], and TooT-BERT-T [7].

Mishra et al. [17] compiled this dataset from the Swiss-Prot database [21]. The initial dataset contained 10,780 well-characterized transporter, carrier, and channel proteins with explicit substrate annotations. They then excluded transporters with more than two substrate specificities, sequences with biological function annotations based only on sequence similarity, and sequences with a similarity greater than 70 % using CD-HIT [22] software. Additionally, they compiled 660 non-transporters as the negative class by randomly sampling proteins from UniProt release 2013_03, excluding the 10,780 transporters. Table 1 presents the final dataset partitioned into training and test sets which is almost balanced between the positive class with 780 sequences and negative class with 600 sequence.

**Table 1:** DS-T: transport proteins dataset.

Class	Training	Test	Total
Transporter	780	120	900
Non-transporter	600	60	660
Total	1380	180	1560

Dataset utilized in this study, derived from the TrSSP project [17]. The dataset comprises two classes, transporters and non-transporters, and is divided into training and test sets.

### 2.2 Protein representation

The field of natural language processing (NLP) has experienced rapid advancements in recent years, driven by attention-based transformers [23], masked language modeling (MLM) [10], and refinements of these methods. Transfer learning and pre-training procedures, utilizing ever-growing datasets, have facilitated the generation of meaningful word and sentence representations [24].

Transfer learning, is the process of applying knowledge acquired from previous tasks to a new task. In deep learning, the prevailing approach for transfer learning involves self-supervised learning on large datasets of unlabeled data. After pre-training, the learned models can be applied to various downstream tasks through fine-tuning with labeled data [25].

#### 2.2.1 BERT

**Figure 3:**
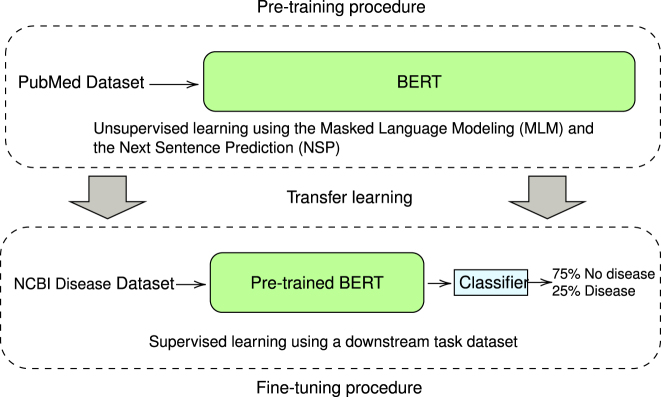
Understanding BERT phases. This diagram depicts two BERT model training steps. (1) Pre-training: unsupervised study of vast amounts of text (articles in PubMed). (2) Fine-tuning: supervised training with a labeled dataset for a particular task.

Google AI researchers developed the Bidirectional Encoder Representations from Transformers (BERT) [10] model for deep contextual language representation. BERT aims to pre-train deep bidirectional representations of words extracted from unlabeled text by simultaneously conditioning on both left and right contexts across all layers [10]. As illustrated in Figure 3, the pre-trained model can be fine-tuned with just one additional output layer to create high-performance models for various downstream tasks, such as text categorization.

BERT [10] is a contextualized word representation model constructed using a Masked Language Model (MLM) and Next Sentence Prediction (NSP) in the pre-training phase. BERT is frequently viewed as a bidirectional strategy since it employs a self-attention mechanism that computes the context utilizing all the terms (left and right) in a sequence.

The task of MLM is to predict the 15 % randomly “masked” tokens. The selected words are not always replaced with the [MASK] token because doing so would create an inconsistency between pre-training and fine-tuning, as the masked token would never be encountered during the fine-tuning phase [10]. Therefore, if the *i*th token is selected, it is substituted with the [MASK] token 80 % of the time. Whereas 10 % of the time is a random token and 10 % of the time, the *i*th token is unchanged [10].

The NSP task is a binary classification problem in which the model takes *sentence pairs* as input and learns to predict whether the second sentence in the pair is the following sentence in the original corpus. This training aim helps grasp the *relationship* between pairs of phrases, which is not directly captured by language modeling but is essential for many downstream tasks, including question-answering (QA) and natural language inference (NLI) [10].

The BERT structure is a multi-layer Transformer encoder [23] in which each encoder layer has an attention mechanism. Two sub-layers form the attention mechanism: multi-head self-attention and feed-forward neural networks. The multi-head self-attention sublayer assists the encoder in monitoring the input text for several words while encoding a single word. Following is the formula for calculating the scaled dot-product attention sublayer [23]:

(1) $$\text{MultiHead}\left(Q,K,V\right)=\text{Concat}\left({\text{head}}_{1},\ldots ,{\text{head}}_{n}\right)W^{o}$$

(2) $${\text{head}}_{i}=\text{Attention}\left(QW_{i}^{Q},KW_{i}^{K},VW_{i}^{V}\right)$$

(3) $$\text{Attention}\left(Q,K,V\right)=\text{softmax}\left(\frac{QK^{T}}{\sqrt{d_{k}}}\right)V$$

where *Q* (Query), *K* (Key), and *V* (Value) are different linear transformations of the input features that offer representations of information for various subspaces. The dimension of *K* is *d*_*k*_, whereas $W_{i}^{Q}$ , $W_{i}^{K}$ , $W_{i}^{V}$ , and $W_{i}^{O}$ are weight matrices.

Two phases comprise BERT: pretraining and fine-tuning. During pretraining, the BERT model is trained on enormous amounts of unlabeled data without supervision utilizing MLM and NSP tasks [10]. Fine-tuning, on the other hand, is the process of initializing the model with pre-trained parameters and then updating those parameters using labeled data from subsequent tasks via an extra classifier [10].

ProtBERT and ProtBERT-BFD comes from ProtTrans project [13] and is based on BERT model [10] that was pre-trained on UniRef100 [14] and BFD [15], a dataset containing 2.1 billion protein sequences, respectively. MembraneBERT [2] is ProtBERT-BFD fine-tuned on the membrane proteins dataset from TooT-M [16].

### 2.3 Traditional classifiers

**Figure 4:**
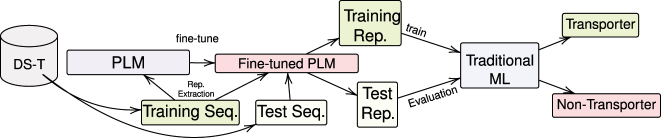
Proposed method of using PLMs and traditional classifiers. Schematic representation of the proposed method for transporter classification, which combines protein language models (PLMs) such as ProtBERT, ProtBERT-BFD, and MembraneBERT with traditional machine learning classifiers to distinguish transporters from non-transporters. The process entails fine-tuning the BERT-based models using the training and validation sets and subsequently extracting representations from the training and test sets to assess the performance of traditional classifiers, including kNN, RF, LR, SVM, and FFNN.

In order to extend the TooT-BERT-T project, we have incorporated several commonly used bioinformatics and membrane protein classifiers [16, 26], namely Logistic Regression (LR) [27], Support Vector Machine (SVM) [28], Random Forest (RF) [29], k-Nearest Neighbor (kNN) [30], and Feed-Forward Neural Network (FFNN)** (see** Figure 4 **)** [31]. The implementations in scikit-learn [32] were utilized for these classifiers.

#### 2.3.1 kNN

kNN is an efficient and simple classification algorithm [33]. The kNN algorithm classifies an unclassified object X according to the class represented by most of its *k* nearest neighbors in the training set vectors. Suppose *k* = 1, the class of object X will be its closest neighbor’s class. The choice of *k* is crucial to the quality of the kNN prediction engine. The hyperparameters that were searched in this study include the number of neighbors (3, 5, 7, 9), algorithm (auto, ball_tree, kd_tree, brute), size of leaf (10, 20, 30, 40, 50).

#### 2.3.2 RF

RF is a well-known technique for machine learning that has lately been successfully applied to various biological prediction issues [34]. RF is a combination of tree predictors such that each tree in the forest depends on the values of a random vector sampled independently and with the same distribution. It has been proven that integrating numerous trees built in randomly selected subspaces can greatly enhance prediction accuracy. RF does a form of cross-validation by utilizing out-of-bag samples. Each tree is formed using a different bootstrap sample from the original data during the training process. A grid search was performed for the RF model with hyperparameters such as the number of estimators (100, 200, 300), minimum samples required to split a node (2, 5, 10), and minimum samples required to be at a leaf node (1, 2, 4).

#### 2.3.3 SVM

SVMs are a potent machine learning tool that is utilized in a variety of biological prediction tools [6]. SVM [35] is an efficient supervised classification technique designed to maximize the margin around the separation boundary between two classes in hyperplanes. In a linear SVM, two line margins are generated and distributed parallel to the two-class data used to generate the margins. About nonlinearly separable data, the kernel trick can be used to transform nonlinear data into a higher-dimensional space in which optimal boundaries can be determined in an efficient, less computationally costly manner than explicit computations of the coordinates. In this study, we performed a grid search of hyperparameters for SVM including C values (0.1, 1, 10, 100), gamma values (1, 0.1, 0.01, 0.001, scale, auto), and kernel types (rbf, sigmoid).

#### 2.3.4 LR

In biological research, LR [36] is a frequently used binary classifier [27]. LR aims to model the linear relationship between the log odd of the positive class and the input variable. The mathematical definition of the relationship is:

(4) $$\ln\left(\frac{p}{1-p}\right)=\beta_{0}+\overset{N}{\underset{i=1}{\sum }}\beta_{i}x_{i}$$

with *p* as the probability the positive class, *x*_*i*_ as the *i*th element of the feature vector, *β*_*i*_ as the coefficient or parameter of *x*_*i*_ (*β*_0_ as the intercept or bias), and *N* as the size of the feature vector. The machine learning algorithm of a logistic regression model aims to discover parameter values that minimize the model’s log loss (a measure of how inaccurate its predictions are). In this study, we evaluated LR with different hyperparameters including the regularization strength (C) with values (0.1, 1, 10, 100), the solver type (newton-cg, lbfgs, liblinear), and the maximum iteration (100, 1000).

#### 2.3.5 FFNN

FFNN [37] is composed of computational unit layers. Connections between units are weighted so that each unit takes the weighted outputs from the previous layer as input. The activation of a particular unit is the weighted total of all inputs to that unit. The activation function determines the output of any unit. The ReLU function is a typical activation function:

(5) f(x)=max(0,x)

The weights of such a network can be optimized, so that specific input patterns correspond to specific output patterns. This is accomplished through the use of a technique known as backpropagation. The network is trained with a collection of corresponding pairs of input and output patterns, beginning with randomly given weights. Hyperparameters are optimized through grid search includes hidden layer size with values [(512, 256, 64, 32, 16), (256, 32), (256)], alpha (0.0001, 0.05), and learning rate (constant, invscaling, adaptive).

### 2.4 Convolutional neural network

**Figure 5:**
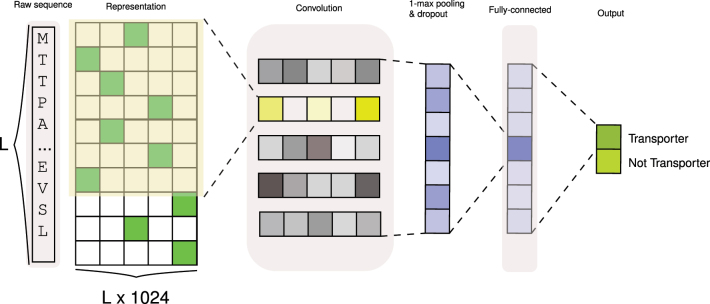
CNN schematic architecture. Workflow of processing sequence representations from PLMs through a CNN neural network. The convolution layer serves as the first layer, followed by 1D max-pooling and dropout. The final layer comprises a fully connected feed-forward neural network, which outputs the probabilities for each class, transporter or non-transporter. “L” denotes the length of the protein sequence.

A Convolutional Neural Network (CNN) [38] is a multilayer neural network (NN) model consisting of convolutional layers followed by fully connected layers. Figure 5 displays the architecture of the CNN network used in this study in which the last layer reflects the class probability of the input proteins. The components are described in the following sections.

#### 2.4.1 Convolution layer

The first layer of our network is a convolutional layer, which can be thought of as a motif scanner [39]. The most often employed convolution type is the 2D convolution layer, abbreviated conv2D. A conv2D layer’s filter or kernel slides through the 2D input data while executing an elementwise multiplication. As a result, the findings will be summed into a single output unit. The kernel will change a 2D matrix of features into a separate 2D matrix of features at each site it traverses.

#### 2.4.2 1-max pooling layer and dropout

The second layer of the proposed CNN is a 1-max pooling layer, one for each convolutional layer. Each of these max-pooling layers only outputs the maximum value of its convolutional layer outputs. The role of this 1-max pooling procedure can be regarded as determining whether or not the motif modeled by the appropriate convolutional layer occurs in the input sequence.

Dropout [40] stochastically eliminates specific neurons during training and has regularization power. We use dropout after the 1-max pooling layer to avoid overfitting and train robust features.

#### 2.4.3 Fully-connected layer

The third layer of the proposed CNN is fully connected. It resembles a conventional feed-forward neural network (FFNN). To classify the protein sequences, a fully connected layer is applied to flattened/concatenated feature vectors.

#### 2.4.4 CNN optimization

First, because all the training data cannot be stored in memory and calculating the training data’s gradients is too time-consuming, we employ the mini-batch gradient descent technique. The loss function is computed as cross-entropy with L2 regularization on the last layer as ProtTrans project [13] in their training computations of the deep models. For an optimization algorithm, we use AdamW optimization [41]. AdamW is a variation of the optimizer Adam [42] with an enhanced weight decay implementation. Regularization is the application of weight decay to reduce the likelihood of overfitting. Adam [42] calculates various and adaptable learning rates for each parameter by utilizing prior gradients and squared gradients, which can mitigate the issue of local optima with stochastic gradient descent. The optimization concludes after ten epochs of training using the training set.

#### 2.4.5 Hyperparameters

The number and length of convolution kernels, the number of perceptrons in the fully connected layer, a coefficient of regularization, dropout rate, learning rate, and batch size are examples of hyperparameters. Since there are too many hyperparameters, exhaustively searching for optimal values for all parameters is impossible. Thus, we use the default settings for most parameters shown to operate well in practice. Five convolutional layers of size 7 were utilized to reduce the embedding to 512, 256, 128, 64, and 32, respectively. During one of the cross-validation sets, the following hyperparameters were set: weight decay = 0.1, dropout rate = 25 %, learning rate = 0.001, batch size = 4, and epoch = 10.

For each classifier (traditional and CNN), using the training set, we found the optimal hyperparameters with 5-fold cross-validation showing in Table 2.

**Table 2:** The selected parameters of the classifiers.

Classifier	Selected parameters
kNN	Algorithm: auto, leaf_size: 10, n_neighbors: 9
RF	min_samples_leaf: 1, min_samples_split: 2, n_estimators: 200
SVM	C: 10, gamma: 0.1, kernel: rbf
LR	C: 1, max_iter: 100, solver: lbfgs
FFNN	Alpha: 0.05, hidden_layer_sizes: (512, 256, 64, 32, 16), learning_rate: constant
CNN	Dropout: 0.25, learning_rate: 3*e* ^−5^, weight_decay: 0.1, epoch: 10

Display of the chosen hyperparameters for each classifier, as determined through a grid search process.

## 3 Training and evaluation

To classify with traditional classifiers, we add a classification layer to the PLMs and train the entire network on the DS-T dataset. The sequences are tokenized using the same pre-trained tokenizer that splits them into amino acids separated by a space. These embeddings are fed as input to the first encoder layer of the BERT model, computing representations from each layer to the end that are passed to the classification layer. The loss of the predicted labels is computed and an optimizer (Adam) is used to minimize the Cross-Entropy loss function, as employed in the original BERT [10] and tested by the ProtTrans project [13]. The fine-tuned representations are extracted using mean-pooling:

(6) $$R_{S}=\text{Mean}{\left(R_{s_{1}}^{1024},R_{s_{2}}^{1024},R_{s_{3}}^{1024},\ldots ,R_{s_{n}}^{1024}\right)}^{D=1024}$$

where Mean calculates the average of all amino acids *s*_*i*_, with $R_{s_{i}}^{1024}$ representing the *i*th amino acid’s representation of the sequence. The length of the sequence is *n*, and each amino acid’s representation is a 1024-dimensional vector. As a result, the sequence’s entire representation, when averaged, has a dimension of *D* = 1024. The extracted representations are input into traditional classifiers for training and evaluation.

**Figure 6:**
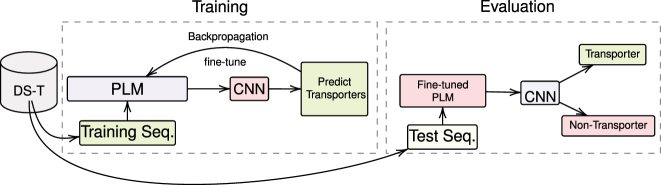
Proposed method for transporters classification using CNN. This figure illustrates the proposed methodology for distinguishing transporters from non-transporters using a deep learning classifier, CNN. The process entails the concurrent training of CNN and fine-tuning of protein language models (PLMs), which include ProtBERT, ProtBERT-BFD, and MembraneBERT.

To fine-tune the PLMs with the CNN classifier (see Figure 6), we used the same approach as explained, but with the CNN added on top of the PLMs without mean-pooling. So the last layer representation from PLM are passed to the first layer of CNN which is a convolution layer and so on. Then, after the classifier layer, the loss is computed using Cross-Entropy and the Adam optimizer is used to discriminate transporters.

We evaluated the efficacy of the predictive model using four widely-used metrics: sensitivity (Sen), specificity (Spc), accuracy (Acc), and Matthews’s correlation coefficient (MCC). The formulas for these metrics are displayed in the table below.

(7) $$\text{Sensitivity}=\frac{TP}{TP+FN}$$

(8) $$\text{Specificity}=\frac{TN}{TN+FP}$$

(9) $$\text{Accuracy}=\frac{TP+TN}{TP+FP+TN+FN}$$

(10) $$\text{MCC}=\frac{\left(TP\times TN\right)-\left(FP\times FN\right)}{\sqrt{\left(TP+FP\right)\left(TP+FN\right)\left(TN+FP\right)\left(TN+FN\right)}}$$

Where *TP*, *TN*, *FP*, and *FN* represent, respectively, the total number of true positive, true negative, false positive, and false negative predictions. Matthew’s Correlation Coefficient (MCC) is a more reliable evaluation statistic for unbalanced data [43].

## 4 Results and discussion

This section analyses the experiment’s outcomes. In the first section, we will analyze the sequence length using a histogram. Then, we assess the output of the conventional machine learning classifiers, followed by an analysis of the outcomes of the deep neural network CNN model. The results show that TooT-BERT-CNN-T, which is the fine-tuned ProtBERT-BFD representation with CNN classifier, outperforms TooT-BERT-T [7].

### 4.1 Sequence analysis

**Figure 7:**
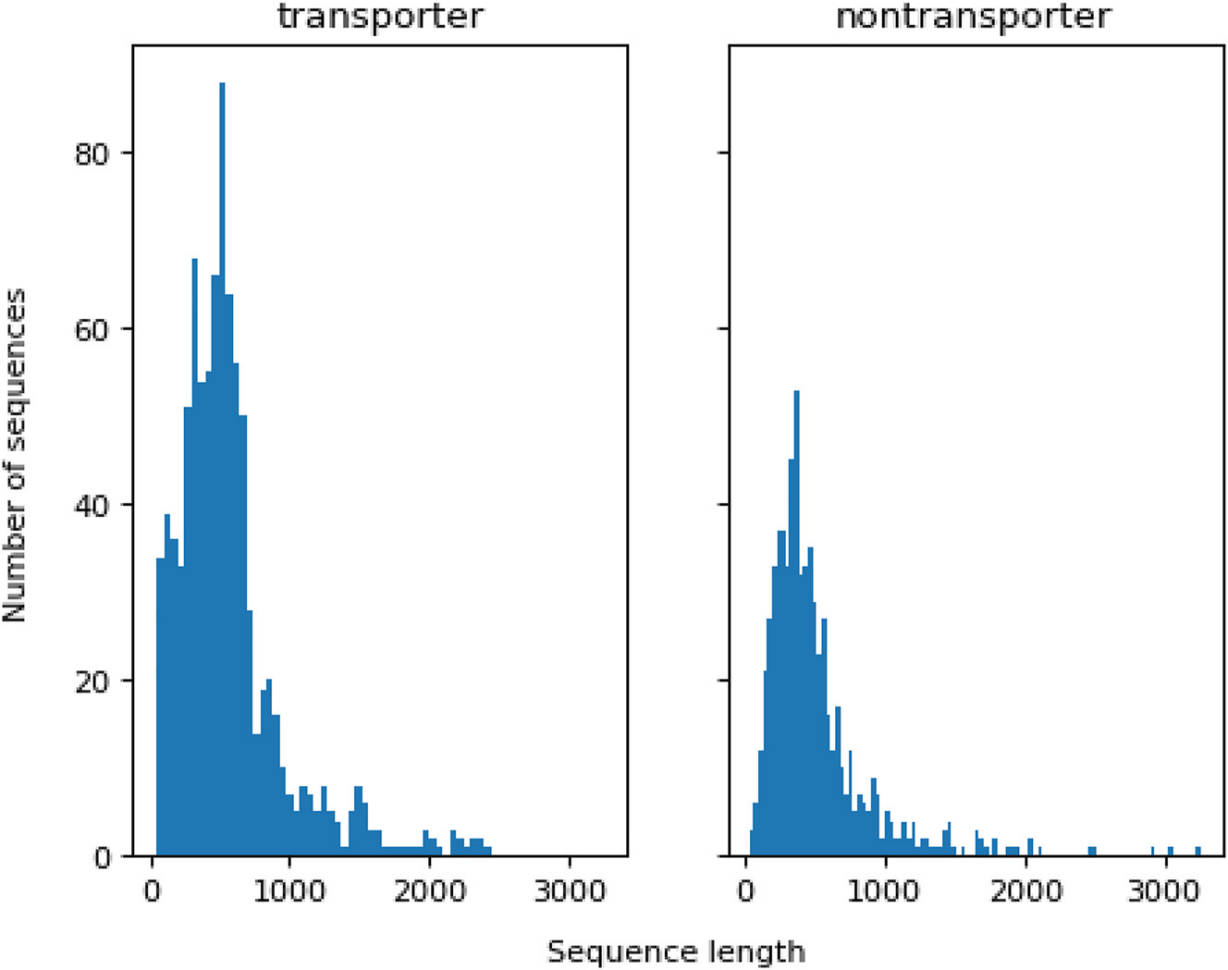
Sequence length distribution. This figure depicts the distribution of transporter (left) and non-transporter (right) sequence lengths.

Our method includes a preprocessing step of truncating protein sequences to a fixed maximum size of 1024, due to memory and computational limitations. When processing the protein sequences, we utilized the tokenizer from the Transformers Python library [44], setting the maximum length parameter to 1024 and enabling truncation. This approach ensured that the tokenizer would automatically truncate the protein sequences at the specified maximum length while retaining the first 1024 amino acids in the event that a given sequence exceeded this length. The histogram of the sequence length of transporters and non-transporters is depicted in Figure 7. The length of a sequence has a substantial effect on its representation to be used in machine learning methods. As shown in Figure 7, most sequences in both classes contain fewer than one thousand amino acids.

### 4.2 Execution time analysis

In this subsection, we provide an analysis of the execution times for both the fine-tuning of ProtBERT-BFD without CNN and with CNN. We acknowledge that the integration of CNNs usually involves a higher consumption of resources and training time. However, as our results indicate, the performance improvement justifies this additional cost.

For the fine-tuning of ProtBERT-BFD without CNN, the training time was approximately 2 h on a Tesla V100 GPU with 32GB RAM. On the other hand, when incorporating the CNN, the training time increased to approximately 2 h and 30 min. This increase in training time can be attributed to the additional complexity introduced by the CNN layers.

### 4.3 LR and SVM surpass the other traditional classifiers

In this section, we will study the findings acquired from classical classifiers. These classifiers were trained using fine-tuned representations of ProtBERT, ProtBERT-BFD, and MembraneBERT.

According to the MCC measure, representations generated from ProtBERT-BFD have demonstrated superior performance compared to alternative representations. This superior performance results from training the model on a more extensive number of sequences during pre-training. Two classifiers, LR and SVM, performed better than other traditional classifiers. The results from CNN are the best in terms of specificity, accuracy, and MCC on the independent test set and among all metrics for CV results, as shown in Table 3.

**Table 3:** Comparison of classifiers using PLM representations.

Classifier	Representation	CV				Independent			
		Sen	Spc	Acc	MCC	Sen	Spc	Acc	MCC
kNN	ProtBERT-BFD	97.02 ± 2.79	97.10 ± 2.78	97.06 ± 2.65	0.9405 ± 0.0537	93.33	88.33	92.20	0.8250
	ProtBERT	91.21 ± 2.37	64.25 ± 2.79	79.49 ± 1.95	0.5857 ± 0.0422	95.00	60.00	83.89	0.6265
	MembraneBERT	98.00 ± 3.54	96.79 ± 5.08	97.47 ± 4.20	0.9485 ± 0.0857	85.83	88.33	86.67	0.7172
RF	ProtBERT-BFD	95.84 ± 3.13	97.11 ± 3.04	96.38 ± 3.08	0.9276 ± 0.0619	94.17	88.33	92.22	0.8250
	ProtBERT	88.40 ± 3.38	76.91 ± 4.40	83.31 ± 2.42	0.6635 ± 0.0493	89.17	78.33	83.89	0.6750
	MembraneBERT	97.82 ± 3.68	96.88 ± 5.10	97.43 ± 4.29	0.9473 ± 0.0877	85.00	90.00	86.67	0.7073
SVM	ProtBERT-BFD	94.05 ± 2.80	86.10 ± 2.68	90.59 ± 2.50	0.7999 ± 0.0506	**100.00**	90.00	92.78	0.8369
	ProtBERT	85.69 ± 2.69	53.97 ± 2.80	71.90 ± 1.64	0.4186 ± 0.0360	**100.00**	86.67	90.00	0.7771
	MembraneBERT	97.65 ± 3.64	96.68 ± 4.81	97.23 ± 4.13	0.9439 ± 0.0838	85.00	91.67	85.00	0.6930
LR	ProtBERT-BFD	96.79 ± 3.27	97.33 ± 2.91	97.03 ± 3.05	0.9400 ± 0.0617	95.83	90.00	93.89	0.8620
	ProtBERT	90.64 ± 2.42	82.33 ± 2.95	87.03 ± 2.02	0.7358 ± 0.0410	92.50	80.00	88.33	0.7347
	MembraneBERT	98.08 ± 3.53	97.00 ± 5.18	97.61 ± 4.25	0.9513 ± 0.0866	86.67	85.00	86.11	0.6989
FFNN	ProtBERT-BFD	92.13 ± 7.08	91.79 ± 6.98	91.79 ± 6.98	0.7924 ± 0.0586	92.50	90.00	90.00	0.8043
	ProtBERT	85.95 ± 6.79	78.44 ± 7.51	82.37 ± 2.29	0.6480 ± 0.0402	**100.00**	50.00	87.22	0.7414
	MembraneBERT	95.37 ± 5.49	94.60 ± 6.73	95.43 ± 4.74	0.9073 ± 0.0936	60.00	28.33	85.00	0.6832
CNN	ProtBERT-BFD	85.64 ± 7.25	95.33 ± 3.85	89.85 ± 3.57	0.8072 ± 0.0642	95.00	**95.00**	**95.00**	**0.8894**
	ProtBERT	95.00 ± 3.58	81.16 ± 1.47	88.98 ± 4.95	0.7855 ± 0.0943	95.00	90.00	93.33	0.8500
	MembraneBERT	98.71 ± 0.90	97.83 ± 1.25	98.33 ± 0.71	0.9662 ± 0.0157	90.83	91.66	91.11	0.8070

This table illustrates the results from three different BERT-based protein representation, namely ProtBERT, ProtBERT-BFD and MembraneBERT with various classifiers. Cross-validation (CV) and independent test set results are presented for each representation and classifier. The maximum value for each column is displayed in boldface.

Using two distinct representations of ProtBERT and ProtBERT-BFD, the performance of the SVM classifier has attained its maximum level of sensitivity, achieving a score of 100 percent on the independent test set. Also, the sensitivity of FFNN with ProtBERT representation on the test set is comparable to the one with SVM.

Regarding accuracy and MCC metrics, LR has the most outstanding values compared to other classical predictors. Moreover, the LR classifier also has the most significant values compared to other traditional classifiers within the CV results. Specifically by MembraneBERT-derived representations. This predictor’s high CV values may suggest that the model has been overfitted to the training set.

As shown in Table 3, the best results on the independent test set for specificity, accuracy, and MCC were achieved by CNN. Also, the CV results from MembraneBERT using the CNN classifier are the highest values among other classifiers.

### 4.4 CNN outperforms the traditional approaches

We have also classified protein sequences using the deep neural network. We evaluated the representations of ProtBERT, ProtBERT-BFD, and MembraneBERT with the proposed CNN architecture.

**Figure 8:**
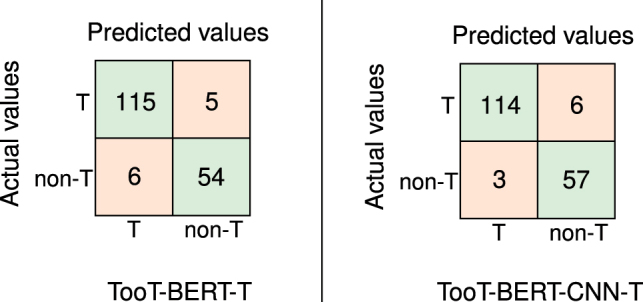
TooT-BERT-CNN-T and TooT-BERT-T confusion matrices. Confusion matrices of TooT-BERT-T and TooT-BERT-CNN-T to discriminate transporters (T) from non-transporters (non-T).

Table 4 and Figure 8 compare the proposed method, TooT-BERT-CNN-T, with previous methods on the independent test set. As can be observed, the performance of TooT-BERT-CNN-T is superior to that of TooT-BERT-T, TooT-T, on three metrics: Specificity, accuracy, and MCC. In comparison, the performance of TooT-BERT-T in terms of the sensitivity measure is superior to that of other classifiers.

**Table 4:** Comparing the classifiers on the independent test set.

Classifier	Representer	Sen	Spc	Acc	MCC
TooT-T [6]	Traditional^a^	94.17	88.33	92.22	0.8200
TooT-BERT-T [7]	ProtBERT-BFD	**95.83**	90.00	93.89	0.8620
TooT-BERT-CNN-T	ProtBERT-BFD	95.00	**95.00**	**95.00**	**0.8894**

TooT-BERT-CNN-T is compared with other classifiers as well as TooT-BERT-T and TooT-T on four evaluation measurements. The maximum value for each column is displayed in boldface. ^a^An ensemble approach of traditional vector representations such as Amino Acid Composition (AAC) and Dipeptide Composition (DPC) [6].

The performance of the proposed method can be attributed to the ProtBERT-BFD-derived representations’ extraction of the network’s superior features. Furthermore, the convolutional filters in CNN’s layers scan the entire feature matrix and perform dimensionality reduction, allowing CNN to perform well in this task, as we believe.

In the following, we outline significant issues that can be addressed in subsequent works. The number of amino acids used in the convolution operation must be specified to employ a CNN deep neural network. This problem, applicable to all k-mer models, is to identify *k* amino acids of a sequence. This experiment utilized *k* = 7 from the ProtTrans [13]. In future tests, *k*’s value might be regarded as a variable that must be evaluated. Furthermore, this study can be used to evaluate the model’s performance in multiclass and multilabel problems of the subtypes of the transport proteins, which is the next objective that can be considered.

## 5 Conclusions

In this experiment, we extended our earlier work on TooT-BERT-T project with the new representation from ProtBERT, which is pre-trained on UniRef100, as well as investigating other classical classifiers and a deep neural network. We exploited the finetuned representation of ProtBERT, ProtBERT-BFD, and MembraneBERT models to train conventional classifiers. In addition, we proposed TooT-BERT-CNN-T, a new structure for CNN deep neural networks, and finetuned the BERT-based models using this network. The obtained results demonstrate that TooT-BERT-CNN-T is superior to conventional models. This significant accomplishment demonstrates that merging two deep neural networks can significantly improve the ability to recognize and analyze the intricate structure of transport proteins. In the future, other deep natural language models from the ProtTrans project can be employed to study and analyze representations of transporters.
